# Supplementary material for: Smart-Home Concept for Remote Monitoring of Instrumental Activities of Daily Living (IADL) in Older Adults with Cognitive Impairment: A Proof of Concept and Feasibility Study
Source: Sensors (Basel). 2022 Sep 7;22(18):6745. doi: 10.3390/s22186745 (PMC9501541; doi:10.3390/s22186745)
Supplement: Supplementary file 1 [file sensors-22-06745-s001.zip › sensors-1866647-supplementary.pdf]

Table S1. IADLSYS Technology Acceptance Model

	Strongly disagree	Disagree	Neither agree nor disagree	Agree	Strongly agree
**Q1:** I think I would need the support of a technical person to be able to use the system.	4	3	2	1	0
**Q2:** Learning to use the system is/was easy.	0	1	2	3	4
**Q3:** The p tags were difficult to work with.	4	3	2	1	0
**Q4:** Wearing the sensors is bothersome.	4	3	2	1	0
**Q5:** Using the system is easy.	0	1	2	3	4
**Q6:** I have concerns about privacy or data sharing on my system.	4	3	2	1	0
